# Swine IFN cocktail can reduce mortality and lessen the tissue injury caused by African swine-fever-virus-infected piglets

**DOI:** 10.3389/fcimb.2024.1388035

**Published:** 2024-12-03

**Authors:** Yitong Jiang, Fei Jiang, Wenzhu Zhai, Ying Huang, Zhongbao Pang, Chunhao Tao, Zhen Wang, Yuheng He, Yuanyuan Chu, Hongfei Zhu, Jiajun Wu, Hong Jia

**Affiliations:** ^1^ Institute of Animal Sciences, Chinese Academy of Agricultural Sciences, Beijing, China; ^2^ China Animal Disease Control Center, Beijing, China

**Keywords:** interferon, cocktail, African swine fever, viral load, mortality

## Abstract

African swine fever (ASF), a highly virulent viral infection, poses a significant threat to the global pig industry. Currently, there are no commercially available vaccines against ASF. While the crucial role of interferon (IFN) in combating viral infections is well-established, its impact on the clinical signs and mortality rates of ASF remains unclear. In this study, swine IFN-α2, IFN-γ, and IFN-λ3 were fused with the Fc segment of immunoglobulin G (IgG) and expressed in mammalian cells (293T), and the antiviral efficacy were detected by VSV-3D4/2 and VSV-PK15 systems. Then, the interferon stimulating genes (ISGs) induced by IFNs-hFc in 3D4/2 cells were determined by qRT-PCR. Also, the preventive potential of the interferon (IFN) cocktail (a mixture of IFNα2-hFc, IFNγ-hFc, and IFNλ3-hFc) were evaluated *in vivo* by 25-day-old piglets. The results showed that the specific activities of IFNα2-hFc, IFNγ-hFc, and IFNλ3-hFc were 2.46 × 10^7^ IU/mL, 4.54 × 10^9^ IU/mL and 7.54 × 10^10^ IU/mL, respectively. The IFN-hFc significantly induced the expression of various IFN-stimulated genes (ISGs) in 3D4/2 cells after IFNs-Fc treatment, including *IFIT5*, *Mx1*, *OASL*, *ISG12*, *STAT1*, *IRF1*, *PKR*, *CXCL10*, and *GBP1*. Furthermore, the IFN cocktail treatment reduced the viral load, delayed death, and reduced tissue injury in the piglets infected with ASF virus (ASFV). in conclusion, these results suggest that the IFNs-hFc showed high anti-viral activity, and the IFN cocktail may be potential for the prevention and treatment of ASF.

## 1 Introduction

African swine fever (ASF) is a highly contagious and lethal viral disease affecting domestic pigs and wild boars, caused by the African swine fever virus (ASFV) (Correi et al., 2013). The mortality rate of acute infections often exceeds 90% (Reis et al., 2017). Presently, there are no commercial vaccines and biological agents for ASF.

Interferon (IFN) plays a crucial role in the host’s natural immunity, defending against pathogenic invasions by stimulating effector cells to produce antiviral proteins (Seo et al., 2011; Zhao et al., 2018). IFNs exhibit broad-spectrum antiviral, antitumor, and immunomodulatory activities (Liu et al., 2019). There are three types of IFNs: Type I IFNs (IFN-α and IFN-β) primarily possess antiviral effects (Schneider et al., 2014). Type II IFN (IFN-γ) mainly regulates the immune function of the body (Schoenborn and Wilson, 2007). Type III IFNs (IFN-λ1, IFN-λ2, IFN-λ3, and IFN-λ4) stimulate the immune system to exhibit antiviral activities (Mesev et al., 2019). Three types of IFNs are functionally synergistic (Sainz and Halford, 2002). Research shows that IFN-α and IFN-γ have significant inhibitory effects against the foot and mouth disease virus (FMDV) and pig reproductive and respiratory syndrome virus (PRRSV) (Su et al., 2013; Shi et al., 2016). However, ASFV has various evolved immune escape strategies to inhibit the host’s antiviral effect and immune response (Zhao et al., 2022). ASFV utilizes its multigene families (MGF360–9L, MGF360–11L, MGF360–12L, MGF360–14L, and MGF505–7R) to bind to interferon-stimulating gene (STING) activators, or directly inhibit key molecules of type I IFN signaling pathway, such as TBK1 and IRF3, and inhibit the expression of type I IFN, thus inhibiting the type I IFNs signaling pathway (Li et al., 2021; Zhang et al., 2022). In addition, proteins like E120R, M1249L, EP364R, C129R, I226R, A137R, I215L, and DP96R are also involved in the regulation of the cGAS-STING pathway to suppress the production of IFNs (Wang et al., 2018; Huang et al., 2021; He et al., 2022). The combined treatment with low-dose IFN-α and IFN-γ (10^5^ IU/kg) not only significantly increases the production of cytokines but also effectively reduces viral load, inhibits ASFV replication, and further reduces early clinical symptoms (Fan et al., 2020). These studies showed that the combination of different types of IFNs, the activation of IFN signaling pathways, and the production of IFN stimulation genes (ISG) may be important strategies to effectively fight against ASFV.

The IFN-hFc enhances the soluble production and robustness of IFN. The hFc domain and its affinity for protein A allow for efficient purification, ensuring high specificity. In targeted delivery, the IFN-hFc fusion protein selectively attaches to antigens on target cell surfaces, enhancing drug buildup in the body and lessening effects on intact tissues. With augmented stability, the IFN-hFc fusion protein more effectively preserves its structural integrity, and augments the protein’s stability and half-life, thereby extending the duration of the drug effect.

In this study, the clinical symptoms, mortality rate, viral load, and tissue injury in pigs were challenged with ASFV following treatment with a cocktail of IFNs, which will provide possibilities for emergency treatment of ASF.

## 2 Materials and methods

### 2.1 Ethics statement

The pig experiment and experimental programs used in this study have been approved by the Ethics Committee of the Chinese Center for Disease Control and Prevention (IAS2022–157). Pig experiments were carried out in the animal biosafety level 3 (ABSL-3) laboratory of the Chinese Center for Disease Control and Prevention.

### 2.2 Cells and virus

Porcine alveolar macrophages (3D4/2) and pig kidney cells (PK15) were purchased from ATCC, China. Human renal epithelial cells (293T) and vesicular stomatitis virus (VSV-GFP) are stored in our laboratory. African swine fever virus (ASFV/CADC_HN09) is preserved in the China Center for Animal Disease Control and Prevention ABSL-3 laboratory.

### 2.3 Expression and purification

According to the gene sequences of pig IFN-α2 (MH538099.1), IFN-γ (NM_213948), IFN-λ3 (NM_001166490.1), and hFc (AF150959.1), the IFN sequences were synthesized and connected with hFc genes. There were no termination codons at the ends of IFN-α2, IFN-γ, and IFN-λ3, and the *Eco*RI enzyme tangent and *Kozak* sequence were introduced upstream of these IFNs; the thrombin lysis site and *Nhe*I enzyme incision point were introduced downstream of the hFc gene; and the codon was optimized according to the preference of mammal codon. The expression plasmid pMal-IFNα2-hFc, pMal-IFNγ-hFc, and pMal-IFNλ3-hFc were transfected into the 293T cells and the cells were cultured for 7 days. The expression of these IFNs was verified using SDS-PAGE, Western Blot, and mass spectrometry. Proteins were purified by using affinity chromatography and were identified by SDS-PAGE and Western Blot, and the protein concentrations were detected by the Bradford method.

### 2.4 Antiviral activity *in vitro*

To assess the antiviral activity of IFNα2-hFc, IFNγ-hFc, and IFNλ3-hFc expressed by mammalian cells, the cytopathic inhibition method of VSV-3D4/2 and VSV-PK15 systems were used *in vitro*. The 3D4/2 cells were prepared into a uniform cell suspension with 1 × 10^6^/mL, then transferred into the 96-well cell culture plate, 100 μL per well, and cultured in a 37°C and 5% CO_2_ incubator for 24 h. All the initial concentrations of the IFNs were 10 μg/μL, and then, they were diluted four times the ratio, totaling eight dilution gradients. Synchronously, blank control, negative control (cells), and positive control (VSV-GFP 100TCID_50_) were set up. Each dilution of IFN-hFc to be tested was added to the corresponding well, 100 μL per well, and each dilution was repeated in six wells, and cultured in 37°C and 5% CO_2_ incubators for 24 h. The liquid was removed and washed twice with PBS; then, 100 μL of 100TCID_50_ VSV-GFP virus solution (1,640 + 2% FBS) was added to each well, and cultured in a 37°C and 5% CO_2_ incubator for 48 h. Once cytopathy (CPE) appeared in positive control wells, the liquid was removed, and washed twice with PBS; the 100 μL of CCK-8 working fluid (10% V/V) was added and cultured in a 37°C and 5% CO_2_ incubator for 2 h, and then, the value of OD_450_ was measured. The antiviral activities of IFNs-hFc were calculated according to the Reed–Muench method (Reed and Muench, 1938).

### 2.5 qRT-PCR analysis for ISGs

To determine the expression of interferon stimulating genes (ISGs) induced by IFNs-hFc (IFNα2-hFc, IFNγ-hFc, and IFNλ3-hFc, at 1 μg/mL) in porcine alveolar macrophages (3D4/2), quantitative real-time polymerase chain reaction (qRT-PCR) was used. The porcine alveolar macrophages (3D4/2), 2 × 10^5^/mL, were grown in a six-cell culture plate, 1 mL per well, and cultivated in a 37°C and 5% CO_2_ incubator for 24 h (set at 0 hours). Then, the cells were treated with IFNs-hFc for 12 h and 24 h, and then, total RNAs were extracted with TRIzol reagent (Invitrogen, Thermo Fisher Scientific, United States), and cDNAs were transcripted with HiScript III RT SuperMix (Vazyme, China Cat. No. R323–01). qRT-PCR was carried out with 2×Color SYBR Green qPCR Master Mix [Vazyme, China (Cat. No. Q712–03)], and detected on the Roche Light Cycler480 (Switzerland) fluorescence quantitative PCR instrument. The 2^-△△CT^ was calculated with the Cycle Threshold (CT value) of the ISG and the beta-actin (β-actin) gene.

### 2.6 Antiviral activity *in vivo*

This study used 25-day-old piglets that were negative for ASFV, classical swine fever virus (CSFV), pseudorabies virus (PRV), porcine parvovirus (PPV), porcine circovirus 1/2 (PCV1/2), porcine reproductive and respiratory syndrome virus (PRRSV), and porcine epidemic diarrhea virus (PEDV). The piglets were randomly divided into three groups (IFN cocktail treatment group, ASFV-infected control group, and PBS cohabitation infection control group), and each group contained three pigs (**Table 1**). They were raised in the ABSL-3 and were intramuscularly injected with 100 HAD_50_ of ASFV/CADC_HN09. The IFN cocktail (100,000 IU/kg per ingredient, 2 mL/pig) was intramuscularly injected 3 days before challenge (-1, -2, -3) and 1, 2, 3, 4, 5, 13, 14, 15, 16, and 17 days post-infection (DPI) in the IFN cocktail treatment group. After the challenge, the body temperature and clinical symptoms were recorded every day in each group, and the mouth, nose, and anal swabs were collected on 0, 3, 7, 11, 14, 18, 21, 25, and 28 DPI. Blood was collected on 0, 3, 7, 14, 21, and 28 DPI. To compare the difference in viral load, the genome DNA of ASFV in the mouth, nose, anal swabs, and blood of pigs in each group after treatment were extracted, and the TaqMan RT-qPCR of the ASFV P72 gene was carried out as described (Fan et al., 2020). Necropsy was performed after death, and tissue was collected for histological examination.

**Table 1:** Grouping and immunization program of the experiment.

Group	Name	Immunization program	Ways to attack drugs	Number of pigs
IFN cocktail treatment group	Compound interferon	-1,-2,-3 days of injection of compound interferon, day 0 cohabitation infection, 1, 2, 3, 4, 5, 13, 14, 15, 16, 17 days of compound interferon injection	Cohabitation infection	3
PBS cohabitation infection control group	PBS	Day 0 cohabitation infection	Cohabitation infection	3
ASFV-infected control group	100 HAD_50_/mL	Day 0 to attack drugs	Intramuscular injection	3

### 2.7 Statistical analyses

Statistical analyses were performed using GraphPad Prism8.0 (GraphPad Software Inc.). Comparisons between groups were performed using the Student’s *t-test*. The data were expressed as the mean ± standard deviation (SD). Differences with *p* < 0.05 were considered statistically significant.

## 3 Results

### 3.1 IFNα2-hFc, IFNγ-hFc, and IFNλ3-hFc exert different antiviral activities

IFNα2-hFc, IFNγ-hFc, and IFNλ3-hFc were purified by protein A chromatographic columns. The concentrations of IFNα2-hFc, IFNγ-hFc, and IFNλ3-hFc were 1.51 mg/mL, 4.3 mg/mL, and 8.1 mg/mL, respectively, as detected by the Bradford method. Meanwhile, the viral inhibitory activity values for IFNα2-hFc, IFNγ-hFc, and IFNλ3-hFc were 2.46 × 10^7^ IU/mL, 4.54 × 10^9^ IU/mL, and 7.54 × 10^10^ IU/mL, respectively, as detected by the VSV-3D4/2 and VSV-PK15 systems (**Table 2**).

**Table 2:** Antiviral activity of IFNα2-hFc, IFNγ-hFc, and IFNλ3-hFc in different cells.

Interferon	Antiviral activity (×10^7^ U/mL) in the cells	
	VSV-3D4/2	VSV-PK15
IFNα2-hFc	2.46	2.64
IFNγ-hFc	454	64.2
IFNλ3-hFc	7,540	81.5

### 3.2 IFNα2-hFc, IFNγ-hFc, and IFNλ3-hFc induce ISG expression in 3D4/2 *in vitro*

**Figure 1:**
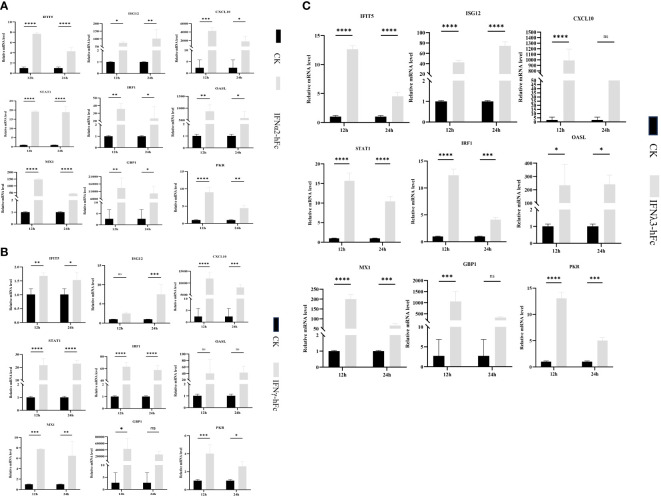
Gene expression in porcine alveolar macrophages (3D4/2) induced by IFNα2-hFc, IFNγ-hFc and IFNλ3-hFc for 12 h and 24 h **(A)** Gene expression in 3D4/2 induced by IFNα2-hFc. **(B)** Gene expression in 3D4/2 induced by IFNγ-hFc. **(C)** Gene expression in 3D4/2 induced by IFNλ3-hFc. It is cultured in the RPMI 1640 medium, including 10% FBS and 5% CO_2_, for 24 h The cells are then incubated with IFNα2-hFc, IFNγ-hFc, and IFNλ3-hFc for 12 h and 24 h, respectively. At 12 h and 24 h, cells are collected and treated with TRIzol reagents to extract RNA. The vertical axis shows the relative transcription level (folding change) of the expression of the IFN stimulating gene (ISG), comparing the negative control group (CK) and the treatment group. The folding changes and normalization of RNA levels were measured by the 2-ΔΔCt method. Three replications were made for the q-PCR verification analysis. The bar represents the mean ± SDs (n = 3). Non-parametric testing is used for difference significance analysis. Statistically significant differences are pointed out (∗*p* < 0.05; ∗∗*p* < 0.01; ∗∗∗*p* < 0.001; and ∗∗∗∗*p* < 0.0001), and the line above the column marks the difference between the two groups.

To investigate whether IFNα2-hFc, IFNγ-hFc, and IFNλ3-hFc fusion proteins induce ISG expression, 3D4/2 cells were treated with IFNα2-hFc, IFNγ-hFc, and IFNλ3-hF for 12 h and 24 h, then, the transcription levels of *IFIT5, Mx1, OASL, ISG12, STAT1, IRF1, PKR, CXCL10*, and *GBP1* were measured via qRT-PCR. The results showed that IFNα2-hFc (**Figure 1A**), IFNγ-hFc (**Figure 1B**), and IFNλ3-hFc (**Figure 1C**) could successfully trigger a range of ISG expressions.

### 3.3 IFNα2-hFc, IFNγ-hFc, and IFNλ3-hFc induce the expression of multiple antiviral genes in 3D4/2 cells

**Figure 2:**
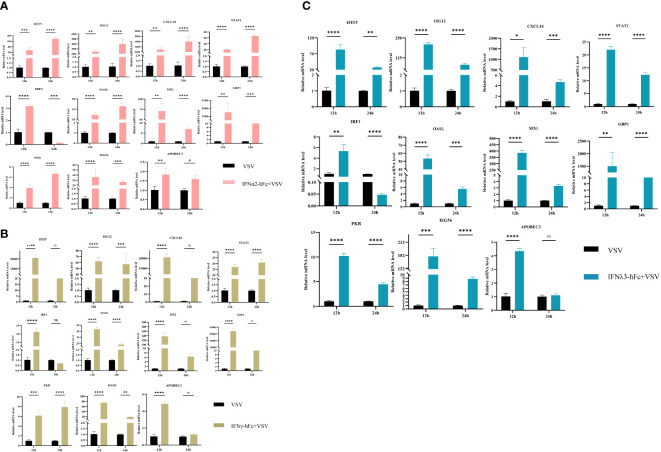
Effects of IFNα2-hFc, IFNγ-hFc, and IFNλ3-hFc on ISG expression after VSV infected with 3D4/2 cells. **(A)** ISG expression after VSV infected with 3D4/2 cells by IFNα2-hFc. **(B)** ISG expression after VSV infected with 3D4/2 cells by IFNγ-hFc. **(C)** ISG expression after VSV infected with 3D4/2 cells by IFNλ3-hFc.Cell samples are collected at 12 h and 24 h The vertical axis shows the relative transcription level (folding change) of the expression of the IFN stimulating gene (ISG), comparing the positive control group (VSV) and the treatment group. VSV challenges the relative transcription level of IFN-stimulating gene (ISG) expression for 12 h VSV challenges the relative transcription level of IFN stimulating gene (ISG) expression for 24 h The folding changes and normalization of RNA levels were measured by the 2-ΔΔCt method. Three replications were made for the q-PCR verification analysis. The bar represents an average of ± SD (n = 3). Non-parametric testing is used for difference analysis. Significant statistical differences are pointed out (∗*p* < 0.05; ∗∗*p* < 0.01; ∗∗∗*p* < 0.001; and ∗∗∗∗*p* < 0.0001), and the line above the column marks the difference between the two groups.

To investigate the antiviral properties of IFNα2-hFc, IFNγ-hFc, and IFNλ3-hFc, 3D4/2 cells were prepared at 2 × 10^5^/mL, cultivated in a six-well plate, 1 mL per well, and cultured in a 37°C and 5% CO_2_ incubator for 24 h. Subsequently, IFNs were added and continuously cultured for 24 h, and then, 100TCID_50_ VSV-GFP were added for 12 h and 24 h. The transcription levels of *IFIT5, Mx1, OASL, ISG12, STAT1, IRF1, PKR, CXCL10, GBP1, ISG56*, and *APDBEC3* genes in 12 h and 24 h were measured using qRT-PCR. The results indicated that IFNα2-hFc (**Figure 2A**), IFNγ-hFc (**Figure 2B**), and IFNλ3-hFc (**Figure 2C**) triggered a variety of antiviral gene expressions combated with VSV infections. The expressions of major antiviral genes such as *Mx1, OASL*, and *PKR*, were higher in 12 h than in 24 h.

### 3.4 IFN cocktail delays death and disease progression in pigs challenged by ASFV

**Figure 3:**
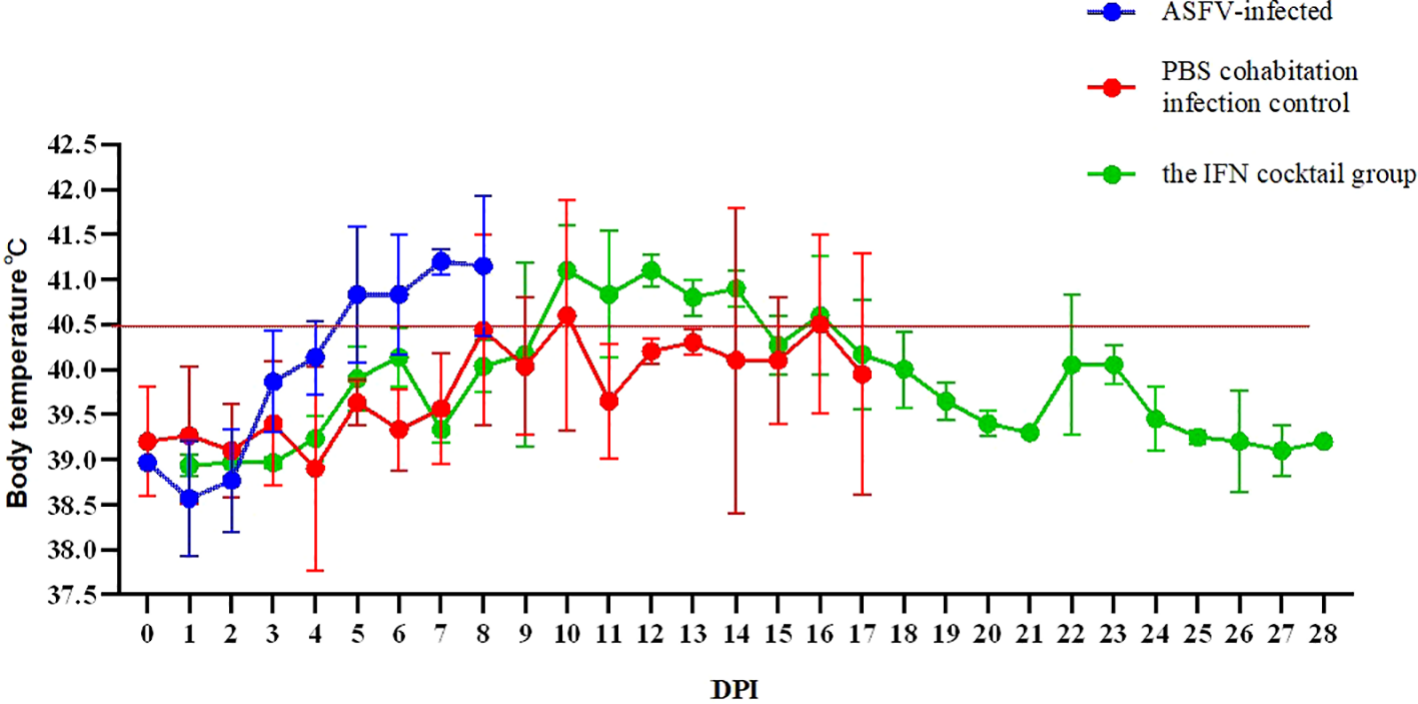
Tests were conducted on the body temperature of pigs 28 days after ASFV infection. In ASFV-infected control group, the peak temperature reached 41.7°C, while the lowest temperature stood at 40.1°C. In PBS cohabitation infection control group, the highest body temperature recorded was 42°C and the lowest temperature was 38.9°C. In the IFN cocktail treatment group, the highest temperature was 41.2°C, and the lowest was 38.8°C, with the temperature of 18 DPI surviving pigs starting to drop from 41°C at 17 DPI.

**Figure 4:**
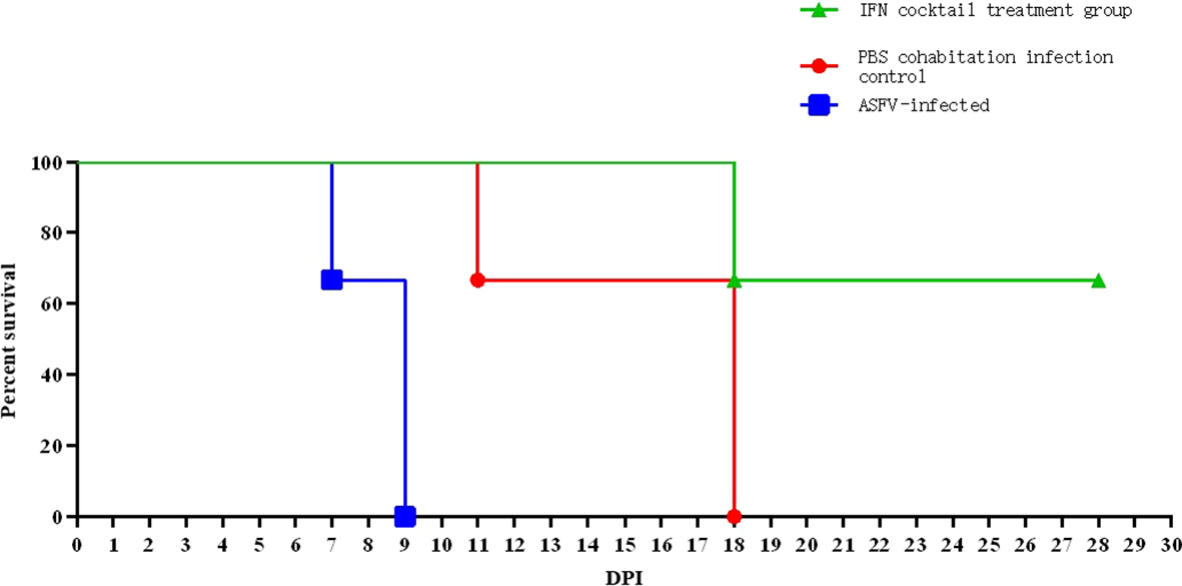
Tests were conducted on the mortality rates of pigs after ASFV infection. In ASFV-infected control group, one pig perished on day 7, achieving a 100% mortality rate on day 9. In PBS cohabitation infection control group, one pig died on day 11 and two pigs died on day 18, resulting in a 100% mortality rate. In the IFN cocktail treatment group, only one pig died on day 18, with a 66.67% survival rate on day 28. Two pigs survived but did not die until 37 DPI.

To examine whether the IFN cocktail can fight ASFV, the pigs were segmented into three distinct groups: the IFN cocktail treatment group, the ASFV-infected control group, and the PBS cohabitation infection control group. After infection, daily temperature (**Figure 3**) and clinical symptoms were monitored. The mortality rates for each category were documented for a period ranging from 0 to 28 days (**Figure 4**). After the ASFV challenge, the temperature of pigs in the ASFV-infected control group increased rapidly to 40.5°C at 3 DPI and reached 41.7°C at 5 DPI, while the lowest temperature stood at 40.1°C. One pig died at 7 DPI, and all pigs died until 9 DPI, with a mortality rate of 100%. The temperature of pigs in the PBS cohabitation infection control group increased to 40°C at 7 DPI and reached the highest of 42°C at 10 DPI and the lowest temperature was 38.9°C. One pig died at 7 DPI, and all pigs died until 18 DPI, with a mortality rate of 100%. Comparatively, the temperature of pigs in the IFN cocktail treatment group increased to 40.5°C at 9 DPI, and reached the highest of 41.2°C at 16 DPI, and the lowest was 38.8°C; one pig died at 18 DPI. Fortunately, the temperature of the other two pigs in the IFN cocktail treatment group dropped from 17 DPI to 28 DPI and survived, with a survival rate of 66.7% until 28 DPI, and died until 37 DPI. The results indicated that the treatment of IFN cocktail could effectively relieve the clinical symptoms of ASF, and delay the death of pigs, and as such, the IFN cocktail could be used as an emergency treatment for ASF.

### 3.5 IFN cocktail treatment can reduce viral load

To further evaluate the effects of interferon cocktail on viral load in the infected pigs, the oral, nasal, and anal swab samples were collected at 0, 3, 7, 11, 14, 18, 21, 25, and 28 DPI, and the genome DNA of ASFV in the mouth, nose, anal swabs, and in the blood of pigs in each group after treatment were extracted, and the TaqMan qRT-PCR of the ASFV P72 gene was carried out, as described (Fan et al., 2020).

**Figure 5:**
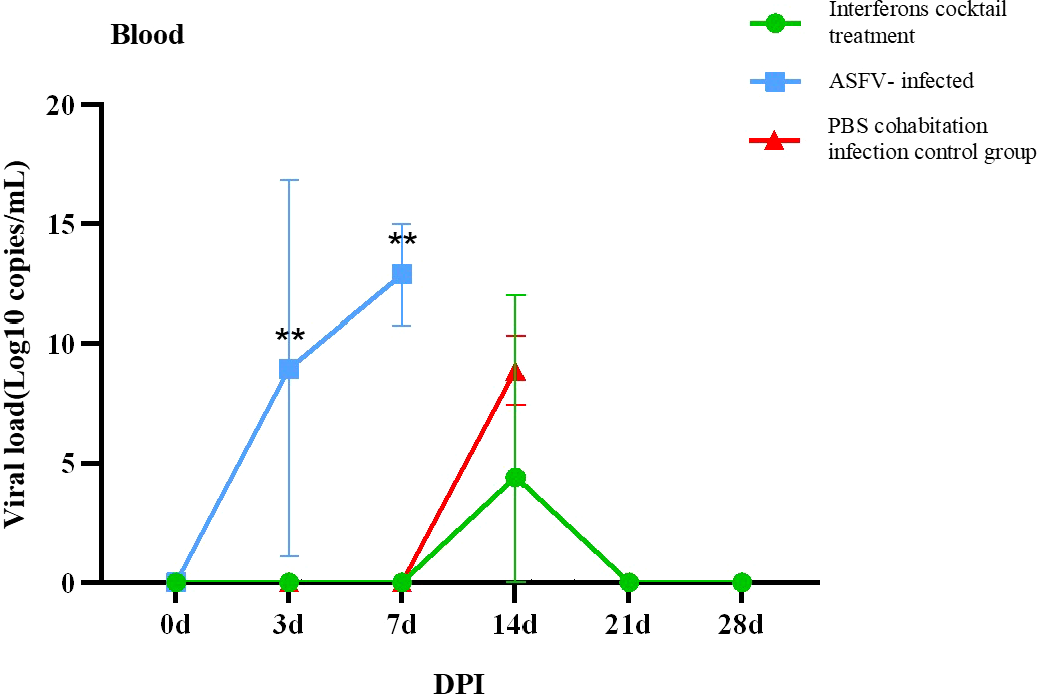
Virus copies of blood post-ASFV challenge. On the third and seventh days, experimental pigs exhibited a notably greater count of ASFV virus copies in their blood compared to those in the interferon treatment and cohabitation infection groups. (∗∗P < 0.01; n=3).

The results (**Tables 3**, **4**) showed that viruses were released in all pigs at 3 DPI. From beginning to death, viruses were released from the oral cavity, nasal cavity, and anus. The viral load of the PBS group cohabitation infection group and ASFV-infected control group was higher than that of the interferon treatment group, and the oral and nasal viral loads of these two control groups were significantly different. Only at 3DPI did the interferon-treated group exhibit higher levels of oral and nasal viruses than the cohabitation infection group. The viral loads in the oral, nasal cavity, and anal regions were detected at 7, 11, 18, and 21 DPI, while the viral loads of the interferon group were lesser than those in the PBS group cohabitation infection group, and ASFV-infected control group, which had notable differences. Two pigs survived in the interferon treatment group. The viral load was detected in the blood of all pigs (**Figure 5**). The results (**Table 5**) showed that during the 3 DPI and 7 DPI tests, two pigs in the ASFV-infected control group had viremia. There was no viremia in the IFN cocktail treatment group except for one pig only at 14 DPI.

**Table 3:** Results of viral load detected by qRT-PCR.

Group	Type	0 day	3 days	7 days	11 days	14 days	18 days	21 days	25 days	28 days
Interferon cocktail treatment group	Mouth	0/3	2/3	0/3	0/3	3/3	1/2	0/2	2/2	2/2
	Nose	0/3	1/3	0/3	0/3	3/3	2/2	0/2	2/2	2/2
	Anal	0/3	0/3	0/3	0/3	1/3	0/2	0/2	0/2	0/2
ASFV-infected control group	Mouth	0/3	2/3	2/2	/	/	/	/	/	/
	Nose	0/3	2/3	2/2	/	/	/	/	/	/
	Anal	0/3	2/3	2/2	/	/	/	/	/	/
PBS cohabitation infection control group	Mouth	0/3	2/3	2/3	2/3	1/2	/	/	/	/
	Nose	0/3	2/3	2/3	2/3	2/2	/	/	/	/
	Anal	0/3	0/3	0/3	1/3	0/2	/	/	/	/

**Table 4:** Viral copies of ASFV in treated pigs (Log_10_ copies/mL).

Group	Type	Sample	0 day	3d	7d	11 days	14 days	18 days	21 days	25 days	28 days
Interferon cocktail treatment group	Mouth	IM-1	0.000	2.864	0.000	0.000	0.000	/	/	/	/
		IM-2	0.000	2.912	0.000	0.000	1.188	0.000	0.000	2.836	7.720
		IM-3	0.000	0.000	0.000	0.000	0.000	3.499	0.000	3.989	7.418
	Nose	IN-1	0.000	2.864	0.000	0.000	0.000	/	/	/	/
		IN-2	0.000	2.912	0.000	0.000	1.188	0.000	0.000	2.836	7.720
		IN-3	0.000	0.000	0.000	0.000	0.000	3.499	0.000	3.989	7.418
	Anal	IA-1	0.000	0.000	0.000	0.000	10.582	/	/	/	/
		IA-2	0.000	0.000	0.000	0.000	0.000	0.000	0.000	0.000	0.000
		IA-3	0.000	0.000	0.000	0.000	0.000	0.000	0.000	0.000	0.000
ASFV-infected control group	Mouth	AM-4	0.000	0.000	/	/	/	/	/	/	/
		AM-5	0.000	4.191	3.163	/	/	/	/	/	/
		AM-6	0.000	5.407	7.285	/	/	/	/	/	/
	Nose	AN-4	0.000	7.892	/	/	/	/	/	/	/
		AN-5	0.000	0.000	6.368	/	/	/	/	/	/
		AN-6	0.000	6.054	12.047	/	/	/	/	/	/
	Anal	AA-4	0.000	10.493	/	/	/	/	/	/	/
		AA-5	0.000	0.000	4.572	/	/	/	/	/	/
		AA-6	0.000	2.455	8.101	/	/	/	/	/	/
PBS cohabitation infection control group	Mouth	PM-7	0.000	0.000	3.302	2.803	1.778	/	/	/	/
		PM-8	0.000	0.000	2.313	9.713	/	/	/	/	/
		PM-9	0.000	0.898	0.000	0.000	0.000	/	/	/	/
	Nose	PN-7	0.000	0.000	0.000	2.699	3.547	/	/	/	/
		PN-8	0.000	0.127	5.500	8.793	/	/	/	/	/
		PN-9	0.000	0.000	0.000	0.000	0.889	/	/	/	/
	Anal	PA-7	0.000	0.000	0.000	0.000	0.000	/	/	/	/
		PA-8	0.000	0.000	0.000	10.747	/	/	/	/	/
		PA-9	0.000	0.000	0.000	0.000	0.000	/	/	/	/

**Table 5:** Viral copies of blood post-ASFV challenge (Log_10_ copies/mL).

Day	Interferon cocktail treatment group			ASFV-infected control group			PBS cohabitation infection control group		
0	0	0	0	0	0	0	0	0	0
3	0	0	0	12.189	0	14.693	0	0	0
7	0	0	0	/	11.364	14.379	0	0	0
14	13.202	0	0	/	/	/	7.850	9.909	/
21	/	0	0	/	/	/	/	/	/
28	/	0	0	/	/	/	/	/	/

### 3.6 IFN cocktail treatment reduces tissue injury

**Figure 6:**
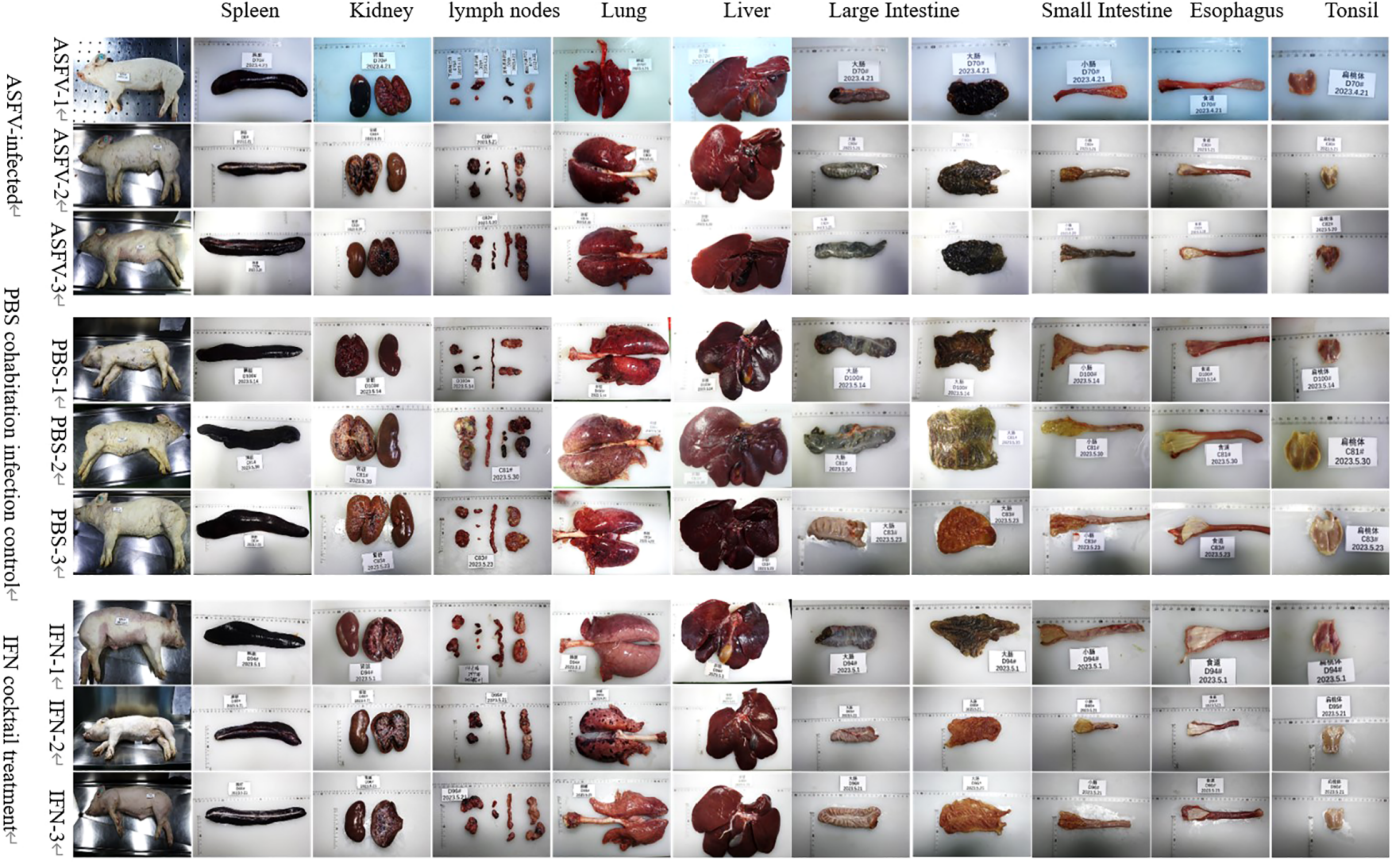
Clinical tissue lesions. The pig spleen, kidneys, liver, pulmonary portal lymph nodes, mandibular lymph nodes, inguinal lymph nodes, mesenteric lymph nodes, and heart from the ASFV-infected control group, the PBS group cohabitation infection group, and the IFN cocktail treatment group all showed typical pathological changes. The degree of lesions in the large intestine, small intestine, and esophagus in the IFN cocktail treatment group was reduced.

To observe whether there are differences in tissue lesions and pathological changes in dead pigs between the IFN cocktail treatment group, ASFV-infected control group, and the PBS group cohabitation infection group, the tissue lesions and the pathological changes of each tissue and organ were observed and recorded including the spleen, kidney, pulmonary portal lymph nodes, mandibular lymph nodes, inguinal lymph nodes, mesenteric lymph nodes, liver, lungs, intestines, esophagus, tonsils, and heart. Clinical tissue lesions (**Figure 6**) showed that the pigs displayed a range of bleeding patterns, encompassing areas on their back, abdomen, limbs, mouth, and nose, accompanied by spleen blockage, growth, lymph node blockage, kidney surface hemorrhage, renal pelvis and calendula hemorrhaging, lung and liver edge blockages, tonsil expansion and blockage, mucous tissue surface hemorrhage, and peripheral fluid accumulation. Compared with the pigs in the ASFV-infected and PBS cohabitation infection control group pigs, tissue damage, tissue swelling, bleeding, and hemorrhagic necrotic spots were significantly reduced by the IFN cocktail treatment. Compared to the ASFV-infected control group and the PBS group cohabitation infection group, the degree of lesions in the intestines and esophagus in the IFN cocktail treatment group was reduced. Only the tonsils of one pig were enlarged and congested, and the tonsils of the other two pigs were not significantly swollen and congested, indicating that the IFN cocktail can reduce the pathological damage of the mucous membrane system of pigs.

**Figure 7:**
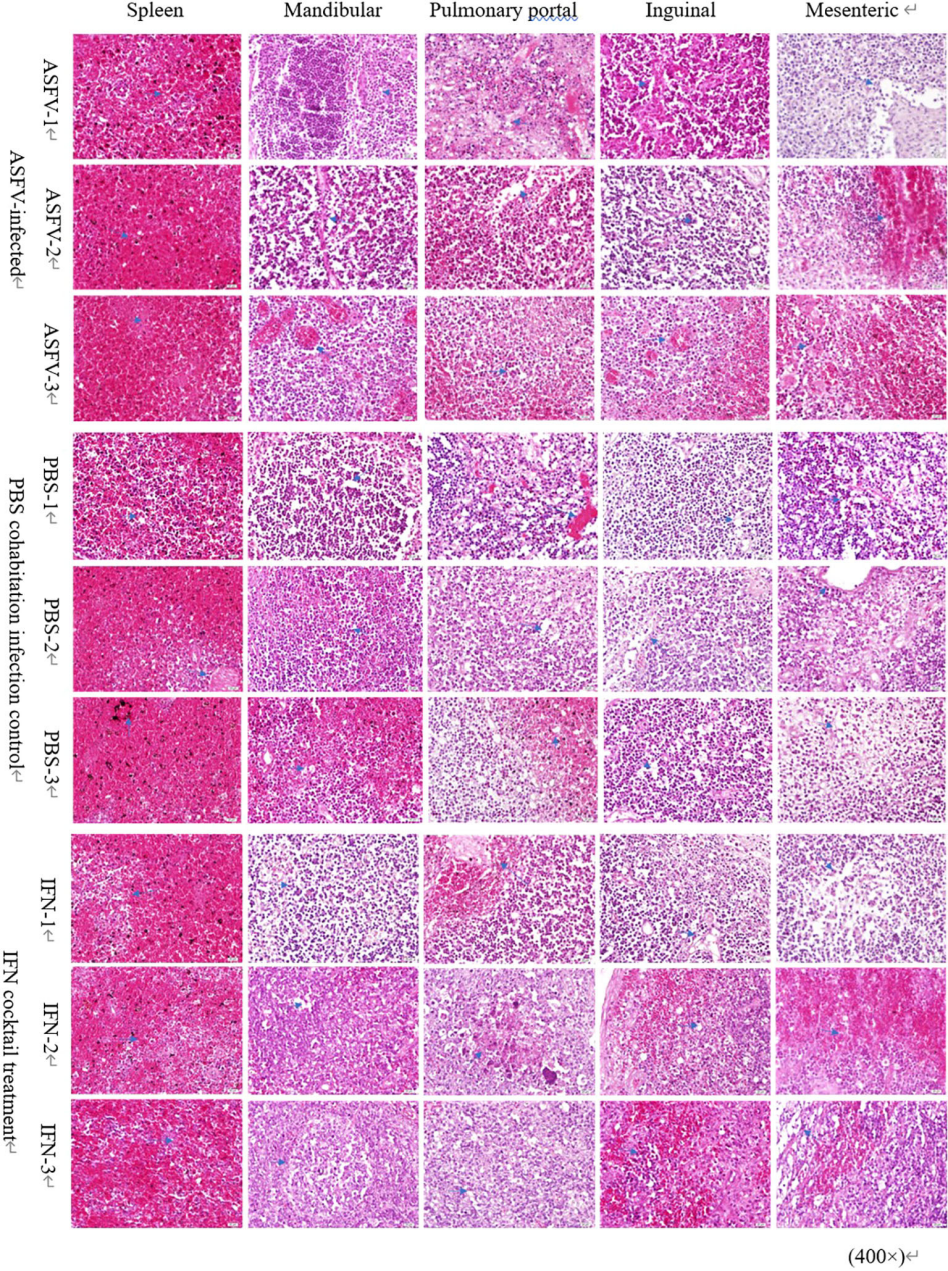
Pathological changes in lymph nodes and spleen. Microscopic examination of sections of the spleen, mandibular lymph nodes, pulmonary portal lymph nodes, inguinal lymph nodes, and mesenteric lymph nodes stained with hematoxylin-eosin (H&E) from the three distinct groups. Arrows mark the pathological alterations observed in the organs. Scale bars represent 100 µm (three fields per pig; three pigs per group).

The analysis and scrutiny of histopathological samples (**Figure 7**) indicated that after the death of pigs in the ASFV infection control, PBS cohabitation infection control, and IFN cocktail groups presented diffused hemorrhage in the spleen tissue, and the lymphocytes in the white pulp have deteriorated, turning necrotic and fragmented, resulting in a discontinuity. The bleeding zone presented an increased quantity of red blood cells and iron-rich hematin. As to lymphatic tissue (mandibular lymph nodes, pulmonary portal lymph nodes, inguinal lymph nodes, and mesenteric lymph nodes) observed in animals after euthanasia, the three distinct groups indicated that the membrane was intact and smooth; the membrane and portal connective tissue extended into the lymph node parenchyma, forming a trabecular structure; the lymphocyte composition was reduced; the lymph node structure was obscure; and the red blood cells around the cortical and medullary lymph nodes were accumulated. There were no obvious differences in tissue damage in the spleen, mandible, hilum, groin, and mesenteric lymph nodes.

## 4 Discussion

ASFV is a highly contagious DNA virus that predominantly replicates in the cytoplasm of infected cells (Reis et al., 2017). It is characterized by acute hemorrhagic fever, and high mortality are the main characteristics of pigs infected with ASFV (Xia et al., 2005). ASFV can inhibit congenital immunity by inhibiting the expression of type I and type II IFN. IFN achieves antiviral effects by inducing different ISGs and improving host immunity (Blome et al., 2013). Studies have shown that the IFN cocktail induces PBMCs to express ISGs *in vitro* and *in vivo*, inhibiting the replication of ASFV, delaying the occurrence of symptoms and disease progression, and reducing tissue damage caused by ASFV infection (Jiao et al., 2023).

Studies show that high-dose ASFV infection inhibits IFN signaling pathways, while low-dose ASFV infection initiates the production of IFNs, and not all infected cells produce IFNs (García-Sastre, 2017; García-Belmonte et al., 2019). Unlike the response of type I IFNs, the reaction of type III IFNs could serve as an initial defense, characterized by its reduced potency, slower progression, and extended duration, thereby causing little inflammatory damage (Lazea et al., 2019; Stanifer et al., 2020). The signaling sequences initiating ISG expression differ between type I and type III IFNs. Both type I and type III IFN utilize JAK1 for signal transmission, while Type I IFN necessitates TYK2 activation, and Type III IFN functions autonomously from TYK2 (Eletto et al., 2016; Catlett et al., 2022). Type III IFN employs JAK2 for signal transmission, unlike Type I IFNs, which function independently from JAK2 (Pervolaraki et al., 2017). Some ISG USP18, known as inhibitory regulators, play a role in managing type I IFN signals, but they are not involved in regulating type III IFN signals. In our study, a cocktail of types I, II, and III IFNs was administered to activate the IFN signaling pathway, thereby amplifying the immune response and ultimately exerting antiviral effects in ASFV-infected piglets (Hoffman and Broderick, 2018).

Several forms of human IgG Fc-fused IFN-α fusion proteins expressed in mammalian cell lines have produced promising results in preclinical studies. Conventional IFN-α, IFN-γ, and IFN-λ3, due to their low molecular weight, can be swiftly eliminated using serum proteolytic enzymes and kidneys. IFN and hFc, a fusion protein, are released into the medium as active homologous dimers linked by disulfide bonds, enhancing the stability of IFNs (Taira et al., 2005). Indeed, over 12 therapeutic Fc fusion proteins, including Enbrel (TNFR-Fc) and Eloctate (Factor VIII-Fc), have received FDA approval for clinical application (Jones et al., 2004; Bitonti and Dumont, 2006; Strohl, 2015). The effectiveness of these instances significantly bolsters our belief in employing Fc fusion protein to enhance the stability of IFNs.

Our study showed that the IFN cocktail could be used as an ASFV emergency treatment preparation and adjuvant treatment to protect pigs from ASF. Enhancing the mammalian expression system during production enabled the acquisition of highly active and stable IFNs. In our study, mammalian cells expressed IFNα2-hFc, IFNγ-hFc, and IFNλ3-hFc, exhibited strong antiviral properties. In 3D4/2 cells, a trio of IFN varieties is capable of triggering diverse gene expressions that stimulate IFNs. Type I IFNs, type II IFNs, and type III IFNs exhibit synergistic functions (Samuel, 2001). To examine whether the IFN cocktail can be anti-ASFV, the pigs were segmented into three distinct groups and challenged with ASFV/CADC_HN09. Within 4 to 6 days after the injection of compound IFNs-Fc, ASFV infection with pig mouth, nose, anal detoxification and viremia were reduced, and so were the clinical symptoms. The degree of the lung, large intestine, small intestine, esophagus, and tonsil lesions in the IFNs-Fc treatment group was reduced, but after death, the spleen and lymph node tissue of the pig was checked in each group. Pathological examination showed that piglets in the ASFV-infected control group, PBS cohabitation infection control group, and IFN cocktail treatment group revealed inflammatory responses in the spleen, mandibular, pulmonary portal, inguinal, and mesenteric lymph nodes. Compared with ASFV-infected control piglets and PBS cohabitation infection control piglets, the piglets that received the IFNs-Fc cocktail treatment exhibited a notable decrease in organ damage and a lower overall inflammatory reaction. There was no obvious difference in pathology, compared with the ASFV-infected control group and PBS cohabitation infection group, the IFNs-Fc cocktail could delay the death of ASFV-challenged pigs by 6 to 19 days. Jiao’s study indicated that although the IFN cocktail treatment did not protect pigs from death after intranasal infection with SY18, it prolonged their survival period after ASFV exposure. Animals in the IFN + SY18 group delayed their death for 5–6 days (Jiao et al., 2023). Compared with their results, our study showed that the IFN cocktail could delay death for 6 to 19 days, which indicated that this IFN-Fc cocktail could further prolong survival after ASFV exposure and protect them for a longer time.

The results indicate that the treatment of IFN cocktail could effectively relieve the clinical symptoms of ASF, and delay the death of pigs. These findings suggest that the treatment with a combination of type I, II, and III IFN in the IFN cocktail improves their efficacy in combating the ASFV challenge, and it is hoped that the IFN cocktail is used as an emergency treatment agent for the prevention and control of ASFV.

## Data Availability

The original contributions presented in the study are included in the article/**Supplementary Material**. Further inquiries can be directed to the corresponding authors.
